# Pharmacokinetics of a New Pharmaceutical Form of Vitamin D3 100,000 IU in Soft Capsule

**DOI:** 10.3390/nu11030703

**Published:** 2019-03-26

**Authors:** Romuald Mentaverri, Jean-Claude Souberbielle, Gilles Brami, Christelle Daniel, Patrice Fardellone

**Affiliations:** 1 Laboratoire de Biologie Humaine, CHU Amiens-Picardie, Biologie Endocrinienne et Osseuse 1, 80 054 Amiens, France; mentaverri.romuald@chu-amiens.fr or romuald.mentaverri@u-picardie.fr; 2 EA 7517, Centre de Recherche Universitaire en Santé de l’UPJV, site Hôpital Sud, 80 054 Amiens, France; fardellone.patrice@chu-amiens.fr; 3 Service des Explorations Fonctionnelles, hôpital Necker-Enfants Malades, 75 015 Paris, France; souberjc@outlook.fr; 4 Laboratoires IPRAD 174/178 quai de Jemmapes, 75 010 Paris, France; g.brami@iprad.com; 5 CHU d’Amiens Picardie Place Victor Pauchet, 80 054 Amiens, France

**Keywords:** vitamin D, single dose, 100,000 IU soft capsule, pharmacokinetics, 25(OH)D, oral supplementation

## Abstract

Vitamin D deficiency is frequent in the general population and both subjects and health professionals could benefit from a broader range of vitamin D3 formulations. We conducted a single-dose, open-label, parallel-group, randomized bioequivalence study to compare a single dose of a newly developed vitamin D3 100,000 IU in a soft capsule (Group 1) with the reference drug vitamin D3 100,000 IU oral solution in ampoule (Group 2) in healthy volunteers over a four-month period. The primary endpoint was the area under the curve (AUC) of serum 25-hydroxyvitamin-D (25(OH)D) concentrations on Day 112. This study was conducted in France from February to June 2014 in 53 young adults with a mean age of 26.9 years. At baseline, low mean serum 25(OH)D levels were observed in both groups (10.6 ng/mL in Group 1 and 9.0 ng/mL in Group 2). On Day 112, the AUC of serum 25(OH)D concentration was 2499.4 ± 463.8 nmol/mL (7.8 ± 0.2 for LogAUC) for Group 1 and 2152.3 ± 479.8 nmol/mL (7.6 ± 0.2 for LogAUC) for Group 2. Bioequivalence of the two treatments was not demonstrated. Superiority of vitamin D3 100,000 IU soft capsule was observed with *p* = 0.029 for AUC and *p* = 0.03 for LogAUC using a non-parametric Wilcoxon test. The profile of the serum 25(OH)D concentration showed a significant difference in favor of Group 1 on Days 1, 3, 7, 14 and 90. Mean serum 25(OH)D concentrations in Group 1 were between 20 and 30 ng/mL during the four-month period and under 20 ng/mL throughout the study in Group 2, except on Day 112. Mean C_max_ for Group 1 was significantly higher (*p* = 0.002). Fourteen days were needed to reach T_max_ by more than half the subjects in Group 1 compared to 45 days in Group 2. Both treatments were well tolerated, with no severe or related adverse events reported. In conclusion, the pharmacokinetic profile of the new formulation of vitamin D3 100,000 IU soft capsule is superior to that of the oral solution in ampoule. The new formulation increased serum 25(OH)D levels to above 20 ng/mL and maintained levels from 20 ng/mL to 30 ng/mL for four months in late winter and spring.

## 1. Introduction

The serum 25-hydroxyvitamin D (25OHD) concentration is the consensual marker of vitamin D status [1]. Vitamin D deficiency has been associated with numerous events and conditions such as falls, fractures, osteoporosis, diabetes, autoimmune disease, cardiovascular and renal diseases, infectious diseases, depression, neurodegenerative diseases, and cancer [1,2].

25-hydroxyvitamin D (25(OH)D) is the main circulating metabolite of vitamin D and its serum concentration is considered a consensual reliable marker of vitamin D status [1]. Most experts consider that a serum 25(OH)D concentration between 20 ng/mL and 50 ng/mL (50–125 nmol/L) is associated with optimal health in the general population [3] and a concentration <12 ng/mL (30 nmol/L) is considered to represent severe vitamin D deficiency. Many other experts also consider that a 25(OH)D concentration of at least 30 ng/mL (75 nmol/L) [4] or in the range of 40–60 ng/mL (100–150 nmol/L) [5] is more appropriate, at least in some patients such as elderly subjects who are at elevated risk of falls and fractures, or those with or at risk of osteoporosis, chronic kidney disease, or suffering from malabsorption. Whatever the 25(OH)D cut-off, vitamin D deficiency/insufficiency is highly frequent. Indeed, it is recognized that approximately 30–50%, and 75–85% of the general, apparently healthy, population in western countries have a 25(OH)D level below 20 ng/mL and 30 ng/mL respectively, probably due to a lack of sunshine for a prolonged period or a lack of sunshine exposure in the more southernly countries, and a lack of governmental policy of vitamin D fortification of foods or vitamin D supplementation (for an extensive review of the vitamin D status worldwide see [6,7]). Even severe vitamin D deficiency (25(OH)D < 10–12 ng/mL) is considered as unacceptably frequent in Europe [8].

This is equally true in France where three large epidemiological studies have examined the vitamin D status of the healthy general population (SUVIMAX, 1994 [9,10]; ENNS, 2006–2007 [11]; VARIETE, 2011–2012 [12]). They reported that the mean 25(OH)D concentration was between 20 ng/mL and 24 ng/mL and that approximately 80%, 40–50% and 10% had a concentration below 30 ng/mL, 20 ng/mL, and 10 ng/mL, respectively. However, when stratifying the results according to the season of blood sampling, the mean concentration was well below 20 ng/mL in the winter months in all three studies.

This was not unexpected as humans acquire the majority of their vitamin D from UVB-induced cutaneous synthesis, (approximatively 80%), and as the UVB are seldom present during winter at the latitude of France [1].

Thus, these studies show that vitamin D insufficiency, and severe vitamin D deficiency, is frequent in the French general population and in the rest of Europe, and the authors call for urgent measures to improve the vitamin D status of the general population, especially by vitamin D supplementation.

As a result of the new knowledge about vitamin D and the widespread insufficiency, governments and medical associations worldwide have produced guidelines for reference daily intake (RDI) of vitamin D necessary to ensure good calcium homeostasis and to prevent the classic bone-related burden of vitamin D deficiency [3,4,5,13,14,15,16,17].

Currently, with the exception of an oral vitamin D3 solution that is administered in drops in newborns (one drop = 300 IU), the only pharmaceutical preparation of vitamin D3 available in France is in the form of oral solution in ampoule (currently 50,000 IU, 80,000 IU, 100,000IU, and 200,000 IU). These ampoules are widely prescribed in France following the publication of recommendations on vitamin D supplementation [18,19].

As, like for any drug/nutrient, the pharmacokinetics of vitamin D may vary according to the vehicle used for its delivery, we considered that it was interesting to compare different galenic forms of a similar dose of vitamin D3.

In this context, a new pharmaceutical form of vitamin D3 100,000 IU in a yellow soft capsule without welding (KIPOS^®^) has been developed. This is an oily solution with 2.5 mg of Cholecalciferol. Here, we present the results of a single-dose, open-label, parallel-group, randomized study to compare the pharmacokinetics of vitamin D3 with the stand-alone vitamin D3 100,000 IU oral solution in ampoule (UVEDOSE^®^) in healthy adult volunteers.

## 2. Materials and Methods

### 2.1. Study Design and Healthy Subjects

This was a bioequivalence, open label, randomized, parallel-group clinical study, conducted in one center in France, comparing a single and oral dose of the newly developed vitamin D3 100,000 IU soft capsule (KIPOS^®^, test drug—Laboratoires IPRAD PHARMA—France) with vitamin D3 100,000 IU oily solution of cholecalciferol (2.5 mg) in ampoule (UVEDOSE^®^, reference drug—Laboratoires CRINEX—France) for four months. The open label nature of the study was required because of the difference between both pharmaceutical forms (soft capsule versus oral solution in ampoule). The study was approved by the Nord Ouest II (2013/25) Ethics Committee and was conducted according to the Declaration of Helsinki, Good Clinical Practice, and local regulation. All participating patients provided written informed consent. The study was registered with the identifier EudraCT number: 2013-002041-10.

The study enrolled male and female adult healthy subjects (between 18 and 45 years of age) with a body mass index (BMI) between 18.5 and 27 kg/m^2^. Enrolled subjects had to have at least 10 h per week of sun exposure and a daily milk intake of at least 500 mL for the two weeks prior to the selection visit. Subjects receiving a vitamin D supplement during the three months before the selection visit or having used medications that could interfere with vitamin D during the two weeks before the selection visit were not included. Patients having medical history like hypercalcemia, renal lithiasis, chronic renal disease, hypo or hyperthyroidism, gut surgery (such as bypass), patients having abnormal biological parameters (liver enzymes, creatinine) were not included.

Subjects were randomized to receive a single dose of either vitamin D3 100,000 IU soft capsule (Group 1) or vitamin D3 100,000 IU oral solution in ampoule (Group 2) under nurse supervision. Follow-up visits were scheduled on Days 1, 3, 7, 14, 28, 45, 90 and 112.

Serum 25(OH)D was measured at each visit by enzyme-linked immunoassay (assay kit total Vitamin D (Vit D)-ADVIA Centaur from Siemens Healthcare Diagnostics SAS, Saint-Denis, France). The detection threshold of serum 25(OH)D was 4.20 ng/mL. Serum calcium was measured on Days 0, 45, 90, 112 and parathyroid hormone (PTH) on days 0 and 112 (both by Siemens Healthcare Diagnostics SAS, Saint-Denis, France).

The subjects completed self-questionnaires (during 7 days before the date of the visit) at the selection visit and after treatment on Days 28, 90 and 112. Diet (weight of fish, number of glasses of milk, number of yoghourts, cheese, eggs, vegetables, bread, fruits, cereals, chocolate (dark or milk) per day or per week, quantity of water per day (mineral or not), smoking (number of cigarettes per day), alcohol (number of glasses of wine, beer…per day), drug consumption, and sun exposure (type of skin, duration of sun exposure) were recorded.

### 2.2. Assessment Parameters

The primary endpoint was serum 25(OH)D level measured from Day 0 to Day 112. The study compared the area under the curve (AUC) of the serum 25(OH)D concentration between the two treatment groups.

The secondary endpoints were:

- the maximal concentration of 25(OH)D (C_max_)
- the time required to reach the maximal concentration (T_max_)
- the total Area Under the Curve (AUC) of the serum concentration of 25(OH)D up to Day 28

All adverse events (AEs) observed by the investigators or reported by the subjects were recorded throughout the follow-up period, along with their severity and potential relationship to study treatment.

### 2.3. Statistical Analyses

The primary outcome criterion was the total AUC of serum concentration of 25[OH]D, calculated by trapezoid rule. At least 24 subjects per group were needed (a total of 48 subjects for the study) to reach a power of 80% with α = 5%, equivalence bounds defined as [−0.223; 0.223] and a variability estimated at 16%.

All statistical tests, performed using the software SAS version 9.4 (SAS Institute, Cary, NC, USA), and confidence intervals (CI) were two-sided with a significance level of α = 5% or α = 10% for primary analysis. Estimates of AUC for the primary criterion and of C_max_ for the secondary criteria are presented with their two-sided 90% CIs. The 90%CI of the difference of LogAUC was calculated using an analysis of covariance model and was compared with equivalence bounds defined [−ln(1.25); ln(1.25)] = [−0.223; 0.223].

If the 90% CI obtained was not included in the equivalence bounds, equivalence was rejected and a switch to superiority was considered using the non-parametric Kruskall-Wallis test. Sensitivity analyses adjusted on the 25[OH]D reference value were performed.

Treatment groups were compared by the Chi-Square test for non-ordinal data and the Kruskall-Wallis test for ordinal data.

## 3. Results

### 3.1. Subject Baseline Characteristics and Disposition

A total of 53 healthy adults were randomized and primarily recruited in the winter season (21 and 22 February 2014) in the North of France (latitude 49.9° N): 27 (50.9%) in Group 1 (vitamin D3 100,000 IU soft capsule: KIPOS^®^) and 26 (49.1%) in Group 2 (vitamin D3 100,000 IU oral solution in ampoule: UVEDOSE^®^). All the subjects completed the study and were analyzed as a unique population (Intention To Treat = Per Protocol.)

In Group 1, 70% of the subjects were female and 30% were male, with a mean age of 26.9 years. In Group 2, 50% of the subjects were female and 50% were male, with a mean age of 26.8 years. Both groups had similar baseline characteristics (Table 1) apart from the sex ratio. The difference in sex ratios between the groups was retrospectively tested and was non-significant (*p* = 0.13). However, there were significantly more women in Group 1 (*p* = 0.03 Z-Test).

A multivariate analysis was performed of log AUC adjusted on sex, age and BMI and they did not affect the bioavailability of vitamin D3 100, 000 IU soft capsule versus vitamin D3 100,000 IU.

Low mean serum 25(OH)D levels of about 10 ng/mL were observed in both groups. Physical examination components (heart rate, temperature, blood pressure and physical exam), hematological parameters and biochemical results were within the normal range for all subjects at baseline.

### 3.2. Primary Endpoint

Four months after administration of a single dose of 100,000 IU Vitamin D3, the AUC of serum 25(OH)D levels was 2499.4 ± 463.8 nmol/ml (7.8 ± 0.2 for LogAUC) in Group 1 and 2152.3 ± 479.8 nmol/ml (7.6 ± 0.2 for LogAUC) in Group 2. The 90% CI of the difference of LogAUC obtained [−0.26; −0.06] was not included in the equivalence bounds [−0.223; 0.223]. Consequently, the bioequivalence of the treatments was not demonstrated.

A non-parametric Wilcoxon test concluded in the superiority of vitamin D3 100,000 IU soft capsule versus vitamin D3 100,000 IU oral solution in ampoule. This superiority was observed for the criteria AUC (*p* = 0.029) and for LogAUC (*p* = 0.03) on Day 112 (Table 2).

Sensitivity analysis (analysis of covariance) adjusted to the reference value of the serum concentration of 25(OH)D on Day 0 and the mean value of the three serum 25(OH)D values on Day 14, Day 7 and Day 0 showed that this factor strongly impacted the model (*p* < 0.001). This analysis demonstrates the influence of the 25(OH)D reference value on the AUC. However, adjustment of pharmacokinetic parameters to the serum 25(OH)D reference value did not alter the findings of the lack of bioequivalence between the two treatment groups and the superiority of the capsule.

### 3.3. Secondary Endpoints

**Figure 1:**
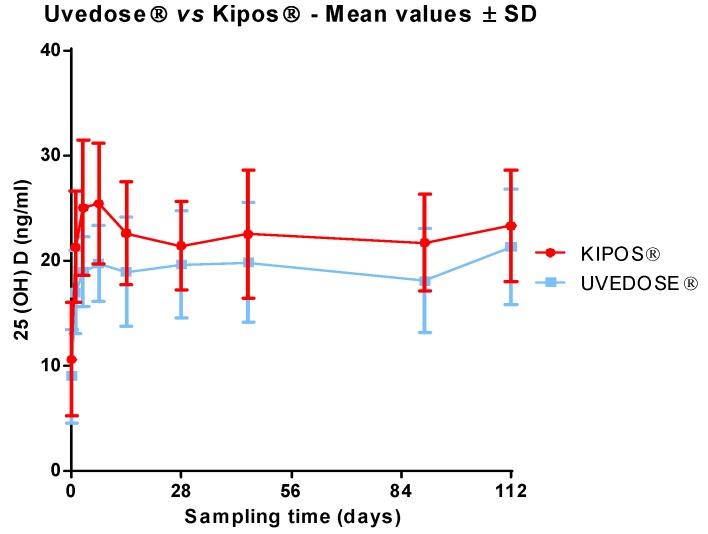
Profile of serum 25(OH)D levels after administration of a single dose of 100,000 UI of vitamin D3.

The profile of the serum 25(OH)D concentration after administration of a single dose of 100,000 UI of vitamin D3 showed that the serum 25(OH)D level in Group 1 was higher than in Group 2 at all time-points (Figure 1). The difference was statistically significant on Day 1 (*p* = 0.002), Day 3 (*p* < 0.001), Day 7 (*p* < 0.001), Day 14 (*p* = 0.019) and Day 90 (*p* = 0.016).

Mean serum 25(OH)D levels in Group 1 were between 20 ng/mL and 30 ng/mL during the four months after administration and under 20 ng/mL in Group 2 until Day 112 (21.28 ng/mL).

Mean C_max_ for Group 1 (28.5 ± 5.0 ng/mL) was significantly higher (*p* = 0.002) than mean C_max_ for Group 2 (23.9 ± 4.3 ng/mL). T_max_ was reached within 14 days by more than half of the subjects who received vitamin D3 100,000 IU soft capsule as opposed to 45 days in Group 2. However, T_max_ value distribution was not statistically different in the two groups (*p* = 0.338).

Superiority of vitamin D3 100,000 IU in soft capsule was also demonstrated for the AUC, LogAUC on Day 28 and for C_max_ using a non-parametric Wilcoxon test.

**Figure 2:**
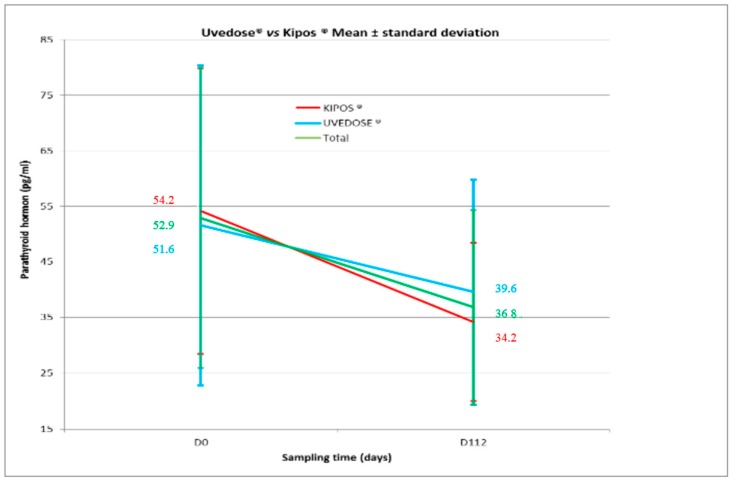
Serum PTH levels after administration of a single dose of 100,000 UI of vitamin D3.

A significant decrease in serum PTH levels was observed in both groups between Days 0 and 112 for Group 1 (−20.0 ± 22.8; *p* < 0.001) and Group 2 (−12.0 ± 23.3; *p* = 0.016) as shown in Figure 2. However, there was no statistical difference between the two groups (*p* = 0.212).

Serum calcium concentration was stable over time and remained the same on Day 45, Day 90 and Day 112 in both groups (2.3 mmol/L) with no statistical difference between groups (*p* = 0.481, *p* = 0.660 and *p* = 0.323, respectively).

Food consumption, sun exposure, tobacco, alcohol and drug consumption were not found to have any impact on the pharmacokinetic parameters (AUC, C_max_ and T_max_) after analysis of data collected from self-reported questionnaires. Treatment effect remained the same as primary analysis.

### 3.4. Safety Analysis

Overall, five (18.5%) of the 27 subjects who received vitamin D3 100,000 IU in soft capsule and seven (26.9%) of the 26 subjects who received vitamin D3 100,000 IU oral solution in ampoule experienced an adverse event (AE). None of the AEs were severe or related to investigational products. No AE led to study discontinuation. No adverse drug reaction was reported.

## 4. Discussion

This study compared the pharmacokinetics of a single dose (100,000 IU) of oral vitamin D3 in soft capsule to the same single dose of vitamin D3 supplied as oral solution in ampoule in healthy subjects in the North of France (latitude 49.9° N) over a four-month period.

Our study was initiated during winter and baseline blood samples showed that the study population was deficient in vitamin D with a mean serum 25(OH)D level below 12 ng/mL in both groups. These findings are consistent with current epidemiologic data reporting a low vitamin D status in European and French populations [8,20]. Seasonal fluctuations of 25(OH)D concentration has been shown to affect the prevalence of insufficiency and deficiency in the literature [21,22,23], and highlights the fact that the season needs to be taken into consideration in the management of vitamin D status.

In our study, a single dose (100,000 IU) of oral vitamin D3 in soft capsule resulted in an increase in serum 25(OH)D level to above 20 ng/mL and serum 25(OH)D levels were maintained at between 20 ng/mL to 30 ng/mL for the four-month study period. However, serum 25(OH)D did not attain the target of 30 ng/mL in either group.

The immediate post-administrative (Day 3) increase in serum 25(OH)D concentration was higher in Group 1 than in Group 2 (*p* < 0.001). There was a rapid increase of 25(OH)D level with a stabilization of the 25(OH)D level over the four-month period contrary to results observed by Valimaki et al. [24] in a population of elderly women in southern Finland (latitude 60–61° north) who received a 100,000 or 200,000 IU vitamin D3 dose every three months, and in whom the serum 25(OH)D concentration decreased after a peak observed seven days after each dose.

The highest 25(OH)D concentration observed in our study was 25.44 ng/mL on Day 7 in one subject of the vitamin D3 100,000 IU soft capsule group. Such a peak of 25(OH)D concentration on Day 7 is much lower than what was reported by Ilahi et al. [25] in a similar pharmacokinetic study in which 30 subjects received a single dose of 100,000 IU vitamin D orally. This is probably due to the much higher 25(OH)D baseline level in the Ilahi study. Also, as in the Valimaki study, the mean 25(OH)D concentration declined approximately linearly after the initial peak concentration in the Ilahi study in contrast to our results showing a stabilization of serum 25(OH)D during the four-month period. The difference may be due to the timing of the blood sampling to measure 25(OH)D in our study—Day 112 was in June and for Ilahi, it was in February (recruitment in October).

We found that the serum 25(OH)D concentrations was negatively correlated with serum PTH levels. Such a decrease in PTH was also shown by Valimaki [24]. There was no change in serum calcium levels.

Our study failed to demonstrate bioequivalence between the two formulations of 100,000 IU of vitamin D3 (KIPOS^®^ 100 000 IU, soft capsule and UVEDOSE^®^ 100,000 IU, oral solution in ampoule). The superiority of KIPOS^®^ over UVEDOSE^®^ was shown for the pharmacokinetic parameters AUC, LogAUC, and C_max_. The non-demonstration of bioequivalence may be due to the different formulations of vitamin D3. While the vitamin D content (100,000 IU) is identical in both formulations, the content of the oral solution in ampoule may not be totally available, even though the tested products were administered under nurse supervision. The oily nature of the solution means that some can remain in the ampoule. In contrast, the pharmaceutical form of the new medicinal product KIPOS^®^ as a capsule for oral use allows complete administration of the full dose.

One of the limitations of the present study is that, despite no evidence of safety issues in terms of serum calcium concentration, we did not measure urinary calcium excretion. In fact, hypercalciuria may precede hypercalcemia in case of vitamin D overdosing. Another limitation can be highlighted: it might have been more appropriate to do a cross-over study with the same subjects after a washout period. This would have diminished the inter-subject variability.

Future studies comparing the pharmacokinetics of other galenic forms, dosages (25,000 IU, 50,000 IU…), and frequency of vitamin D are warranted in order to learn what steps are appropriate to achieve at least 30 ng/mL 25(OH)D.

## 5. Conclusions

This study highlights that severe vitamin D deficiency is highly prevalent in a population of healthy young adults living in the north of France, supporting the need for supplementation.

The pharmacokinetic profile of the new formulation of vitamin D3 100,000 IU soft capsule KIPOS^®^ was found to be superior to that of the oral solution in ampoule. A single dose of 100,000 IU vitamin D3 in soft capsule KIPOS^®^ resulted in an increase in serum 25(OH)D level to above 20 ng/mL and a level of between 20 to 30 ng/mL was maintained throughout the four months. Vitamin D status in subjects, who were deficient at baseline, improved. Nevertheless, serum 25(OH)D levels did not reach the recommended vitamin D status (30 ng/mL 25(OH)D threshold) by many experts, suggesting that, at least, another intake of 100,000 IU could be necessary to reach this level.

The new formulation of vitamin D3 was well tolerated. This new galenic form in capsule offers a new perspective in the management of vitamin D deficiency, ensuring administration of an optimal dose of vitamin D3.

## Figures and Tables

**Table 1:** Characteristics of participants.

Variables	Group 1 (n = 27) Mean ± Standard Deviation (SD)	Group 2 (n = 26) Mean ± SD
Sex (%Female and 95% CI)	19 (70.4% [53.1%; 87.6%])	13 (50.0%: [30.8%; 69.2%])
Age (year)	26.9 ± 5.9	26.8 ± 5.8
Height (cm)	170.0 ± 8.6	171.3 ± 9.3
Weight (kg)	64.5 ± 9.1	67.7 ± 10.6
Body Mass Index BMI (kg/m^2^)	22.3 ± 2.0	23.0 ± 2.2
25(OH)D (ng/mL)	10.2 ± 5.7	9.6 ± 4.9
Calcium (mmol/L)	2.3 ± 0.1	2.3 ± 0.1
Parathyroid hormone (pg/mL)	54.2 ± 25.7	51.6 ± 28.7

**Table 2:** Area Under the Curve (AUC) and log AUC at Day 112.

	Group 1 (n = 27) Mean ± SD	Group 2 (n = 26) Mean ± SD	*p*-Value ^1^
AUC on Day 112 (nmol.mL)	2499.4 ± 463.8	2152.3 ± 479.8	0.029
Log AUC on Day 112 (nmol.mL)	7.8 ± 0.2	7.6 ± 0.2	0.03

^1^ Wilcoxon non-parametric test.

## References

[1] Holick M.F. (2007). Vitamin D Deficiency. N. Engl. J. Med..

[2] Plum L.A., DeLuca H.F. (2010). Vitamin D, disease and therapeutic opportunities. Nat. Rev. Drug Discov..

[3] Ross A.C., Manson J.E., Abrams S.A., Aloia J.F., Brannon P.M., Clinton S.K., Durazo-Arvizu R.A., Gallagher J.C., Gallo R.L., Jones G. (2011). The 2011 Report on Dietary Reference Intakes for Calcium and Vitamin D from the Institute of Medicine: What Clinicians Need to Know. J. Clin. Endocrinol. Metab..

[4] Holick M.F., Binkley N.C., Bischoff-Ferrari H.A., Gordon C.M., Hanley D.A., Heaney R.P., Murad M.H., Weaver C.M., Endocrine Society (2011). Evaluation, treatment, and prevention of vitamin D deficiency: An Endocrine Society clinical practice guideline. J. Clin. Endocrinol. Metab..

[5] Pludowski P., Holick M.F., Grant W.B., Konstantinowicz J., Mascarenhas M., Haq A., Povoroznyuk V., Balatska N., Barbosa A.P., Karonova T. (2018). Vitamin D supplementation guidelines. J. Steroid Biochem. Mol. Biol..

[6] Hilger J., Friedel A., Herr R., Rausch T., Roos F., Wahl D.A., Pierroz D.D., Weber P., Hoffmann K. (2014). A systematic review of vitamin D status in populations worldwide. Br. J. Nutr..

[7] Mithal A., Wahl D.A., Bonjour J.-P., Burckhardt P., Dawson-Hughes B., Eisman J.A., El-Hajj Fuleihan G., Josse R.G., Lips P., Morales-Torres J. (2009). Global vitamin D status and determinants of hypovtaminosis D. Osteoporos Int..

[8] Cashman K.D., Dowling K.G., Škrabáková Z., Gonzalez-Gross M., Valtueña J., De Henauw S., Moreno L., Damsgaard C.T., Michaelsen K.F., Mølgaard C. (2016). Vitamin D deficiency in Europe: Pandemic?. Am. J. Clin. Nutr..

[9] Chapuy M.C., Preziozi P., Maamer M., Arnaud S., Galan P., Hercberg S., Meunier P.J. (1997). Prevalence of vitamin D insufficiency in an adult normal population. Osteoporos Int..

[10] Touvier M., Deschasaux M., Montourcy M., Sutton A., Charnaux N., Kesse-Guyot E., Assmann K.E., Fezeu L., Latino-Martel P., Druesne-Pecollo N. (2015). Determinants of vitamin D status in Caucasian adults: Influence of sun exposure, dietary intake, sociodemographic, lifestyle, anthropometric, and genetic factors. J. Investig. Dermatol..

[11] Vernay M., Sponga M., Salanave B., Oléko A., Deschamps V., Malon A., Castetbon K. (2012). Statut en vitamine D de la Population Adulte en France: l’Etude Nationale Nutrition Santé (ENNS 2006-2007). BEH.

[12] Souberbielle J.C., Massart C., Brailly-Tabard S., Cavalier E., Chanson P. (2016). Prevalence and determinants of vitamin D deficiency in healthy French adults: The VARIETE study. Endocrine.

[13] Haq A., Wilamawansa P., Pludowski P., Al Anouti A. (2018). Clinical practice guidelines for vitamin D in the United Arab Emirates. J. Steroid Biochem. Mol. Biol..

[14] Braegger C., Campoy C., Colomb T., Decsi T., Domellof M., Fewtrell M., Hojsak I., Mihatsch W., Molgaard C., Shamir R. (2013). ESPGHAN committee on nutrition. Vitamin D in the healthy pediatric population. J. Paediatr. Gastroenterol. Nutr..

[15] German Nutrition Society (DGE) (2012). New reference values for vitamin D. Ann. Nutr..

[16] Rizzoli R., Boonen S., Brandi M.L., Bruyere O., Cooper C., Kanis J., Kaufman J.M., Ringe J.D., Weryha G., Reginster J.Y. (2013). Vitamin D supplementation in elderly or postmenopausal women: A 2013 update of the 2008 recommendations from the European Society for Clinical and Economic Aspects of Osteoporosis and Osteoarthritis (ESCEO). Curr. Med. Res. Opin..

[17] Amer Geriatrics Soc Workgroup (2014). Recommendations abstracted from the American Geroatrics Society consensus statement on vitamin D for prevention of falls ad their consequences. J. Am. Geriatr. Soc..

[18] Benhamou C.L., Souberbielle J.C., Cortet B., Fardellone P., Gauvain J.-B., Thomas T., pour le Groupe de recherche et d’information sur les ostéoporoses (GRIO) (2011). La vitamine D chez l’adulte: Recommandations du GRIO. Presse Med..

[19] Vidailhet M., Mallet E., Bocquet A., Bresson J.-L., Briend A., Chouraqui J.-P., Darmaun D., Dupont C., Frelut M.-L., Ghisolfi J. (2012). Vitamin D: Still a topical matter in children and adolescents. A position paper by the Committee on Nutrition of the French Society of Paediatrics. Arch. Pediatr..

[20] González-Gross M., Valtueña J., Breidenassel C., Moreno L.A., Ferrari M., Kersting M., De Henauw S., Gottrand F., Azzini E., Widhalm K. (2012). Vitamin D status among adolescents in Europe: The Healthy Lifestyle in Europe by Nutrition in Adolescence study. Br. J. Nutr..

[21] Thuesen B., Husemoen L., Fenger M., Jakobsen J., Schwarz P., Toft U., Ovesen L., Jørgensen T., Linneberg A. (2012). Determinants of vitamin D status in a general population of Danish adults. Bone.

[22] Oberg J., Jorde R., Almås B., Emaus N., Grimnes G. (2014). Vitamin D deficiency and lifestyle risk factors in a Norwegian adolescent population. Scand. J. Public Health.

[23] Gill T.K., Hill C.L., Shanahan E.M., Taylor A.W., Appleton S.L., Grant J.F., Shi Z., Dal Grande E., Price K., Adams R.J. (2014). Vitamin D levels in an Australian population. BMC Public Health.

[24] Välimäki V.V., Löyttyniemi E., Pekkarinen T., Välimäki M.J. (2016). How well are the optimal serum 25OHD concentrations reached in high-dose intermittent vitamin D therapy? a placebo-controlled study on comparison between 100 000 IU and 200 000 IU of oral D3 every 3 months in elderly women. Clin. Endocrinol..

[25] Ilahi M., Armas L.A., Heaney R.P. (2008). Pharmacokinetics of a single, large dose of cholecalciferol. Am. J. Clin. Nutr..

